# Reliability and concurrent validity of TRAZER compared to three-dimensional motion capture

**Published:** 2021-01-25

**Authors:** Jennifer A. Hogg, Lynette M. Carlson, Abigail Rogers, Mason W. Briles, Shellie N. Acocello, Gary B. Wilkerson

**Affiliations:** ^1^Department of Health and Human Performance, The University of Tennessee Chattanooga, Chattanooga, TN, USA; ^2^Intercollegiate Athletics, University of Missouri, Columbia, MO, USA; ^3^Emory Sports Medicine Center, Emory Healthcare, Atlanta, GA, USA

**Keywords:** kinect, virtual reality, functional performance, reactive agility

## Abstract

**Background:**

Efficient neural processing of visuospatial and proprioceptive input appears to be crucial for avoidance of sport injury. As such, clinically-feasible tests are needed to identify deficiencies found by advanced neuroimaging and electrophysiological tests. Three-dimensional motion capture in a laboratory setting is currently the gold standard for measurement of human movement parameters but is costly and requires extensive training. Non-immersive virtual reality systems with body motion tracking, such as TRAZER, may provide a clinically-feasible and portable means of acquiring similar variables. Test-retest reliability and concurrent validity of these systems are currently lacking.

**Aim:**

The aim of the study was to assess the concurrent validity of the TRAZER single-camera system with 3D motion capture system and to assess the test-retest reliability of TRAZER’s whole-body reactive agility metrics.

**Methods:**

Participants – For validity, 13 healthy individuals (24.8±3.1 years, 170.0±7.7 cm, 70.0±14.2 kg); for reliability, 18 healthy individuals (23.3±2.5 years, 168.2±11.2 cm, 78.2±17.8 kg). Design – Validity was a single-session cross-sectional study. Reliability was a 3 consecutive day test-retest study. Setting–Controlled laboratory study. Intervention – Assessments utilized randomized movements in eight directions for forty total repetitions as designated by the TRAZER system. TRAZER protocol was simultaneously tracked by Vicon Motion Capture and the TRAZER system. Reliability data were captured on three consecutive days by the TRAZER system. Main Outcome Measures – Maximum acceleration, maximum velocity, and total distance were recorded for validation. In addition to these measures, maximum deceleration, average velocity, average acceleration, average deceleration, and average reaction time were collected for reliability.

**Results:**

Overall, a lack of agreement exists between maximum outputs for TRAZER and 3D motion capture (velocity r=0.808, acceleration r=−0.090), but total distance correlation was high (r =.961). ICC values between days 1-2-3 for average measures were high (average velocity=0.847, average acceleration=0.919, and average deceleration=0.948) with the exception of average reaction time being fair (ICC=0.536). ICCs for maximum measures showed a much smaller correlation between days (velocity=0.654, acceleration=0.171, and deceleration=0.416).

**Conclusions:**

Even though there is a lack of strong concurrent validity between measures obtained from TRAZER and 3D motion capture systems, there is strong test-retest reliability of the TRAZER system. The applicability of these findings makes TRAZER clinically relevant in scenarios requiring pre- and post-testing for return to play decisions, or monitoring of a training regimen where demonstration of validation to a gold standard measurement is not relevant.

**Relevance for patients:**

When test-retest capability is desired, such as in return-to-play protocols following an injury, Trazer is a reliable option.

## 1. Introduction

Functional movement assessments are used by sports medicine clinicians to identify movement dysfunction for purposes of injury prevention, evaluation, rehabilitation, and return to activity decisions. Movement screenings, along with proper corrective interventions, are essential components of injury prevention [1]. Prevention strategies are critical to enhance physical performance, optimize health, minimize health-care expenses, and avoid chronic dysfunction and disability [2]. Portable and convenient acquisition of accurate movement assessment data is necessary for effective injury risk identification and ultimate prevention of sport-related injury.

Lab-based three-dimensional (3D) motion capture systems are the gold standard in functional movement analysis, with reported excellent reliability (ICC_3,k_ > 0.93) [3] and validity (±0.198 mm) (Vicon.com). However, they have limited clinical application due to financial, spatial, and temporal costs [4] and expertise needed to collect and interpret data. Other means of assessing quality of human movement have been developed and studied extensively, including the Functional Movement Screen [5], Star Excursion Balance Test [6], and the Landing Error Scoring System [7]. These tools, however, lack ecological validity, as their required movements are anticipated, whereas responding to unanticipated events and simultaneous performance of cognitive and motor tasks are typically required during athletic activities. Unanticipated events can lead to sensory prediction errors and improper muscle co-contractions, potentially resulting in musculoskeletal injury. Identifying deficits in the simultaneous processing of environmental stimuli and task constraints and in the ability to preplan correct motor sequences (feed-forward) is important for injury prevention [8-10]. Virtual reality systems that track body movements in response to visual stimuli may be valuable for assessing neuromechanical responsiveness, or the ability to optimally integrate neurocognitive and neuromuscular processes [11], and integrated perception-motor neural processes [12], which appear to be crucial for preventing athletic injuries [11].

Virtual reality is an emerging multidisciplinary tool [13] and is typically categorized as either immersive or non-immersive [14,15]. An immersive virtual reality system is one in which the user dons a head-mounted display to interact with their environment, while non-immersive virtual reality is interacted with through a television or computer monitor. TRAZER (Traq Global Ltd, Westlake, OH) is a commercially available non-immersive virtual reality system that utilizes a camera and software gesture recognition. It is marketed as a tool for prevention, rehabilitation, balance training, fall prevention, and concussion management. Its novelty lay in its output metrics. Namely, the measures of total distance, maximum velocity, maximum acceleration, and reaction time are indicative of overall functional performance and as such, may be useful for clinicians and coaches aiming to determine one’s ability to return to sport or their improvement in overall function [11,16].

Briefly, the TRAZER system is a non-immersive virtual reality system that utilizes a Kinect camera. The system employs an infrared camera to create a two-dimensional (2D) representation on a video monitor whereby a participant responds to visual stimuli and is recorded by a Kinect camera. All data captured by the TRAZER system are processed by embedded proprietary algorithms, making it unique to a stand-alone Kinect camera. The Kinect camera has been evaluated for reliability and validity compared to 3D motion capture systems during postural control and balance tasks [17-19], dynamic side-cut maneuvers [20], squatting [21], and single leg squatting [22]. Studies report good to excellent concurrent validity for kinematic angles during a postural balance task (Pearson’s *r*>0.90) [18], (ICC>0.75) [17], side-cutting maneuvers (absolute agreement ICC range=0.77–0.99) [20], and squatting (Pearson’s *r*>0.55) [21]. Test-retest reliability has variable results, from excellent reliability (ICC>0.90) reported by Schmitz *et al.*, 2015 [21], to modest reliability (ICC≥0.70) reported by Clark *et al.*, 2015 [19]. Although Kinect systems have been assessed for accuracy, the accuracy of proprietary algorithms overlaid on Kinect, such as those provided by TRAZER, has yet to be determined.

One study compared TRAZER outputs with 3D Vicon motion capture system during balance and lateral shuffling tests [23]. Three separate and successive trials of four movement patterns were completed by one female and one male participant during simultaneous TRAZER and Vicon captures. The two systems yielded highly correlated results for joint coordinate positions (ICC range=0.75–0.99), joint marker distances (ICC range=0.93–1.0), marker velocities (ICC range=0.93–1.0), joint marker accelerations (ICC range=0.83–0.96), and joint marker decelerations (ICC range=0.91–0.97) [23]. However, due to the small sample size (*n*=2) and single day data collection, there is a need for further investigation.

Therefore, the present study assessed the test-retest reliability of the TRAZER system and its concurrent validity against a 3D Vicon motion capture system. We hypothesized that TRAZER would display moderate test-retest reliability and good to excellent concurrent validity. This study represents a logical step towards the ability to make an informed decision about the fidelity of TRAZER’s measurement of functional movement and its use in the clinic and the laboratory. If TRAZER demonstrates agreement against the gold standard 3D motion capture, the utilization of a more clinically-feasible alternative may expand clinicians’ resources. The ability to accurately measure movement through a commercially available system has the potential for widespread adoption. Reliability is important in the clinic as well as in the laboratory, as reproducing consistent outcomes allow for accurate comparisons of patient progress.

## 2. Methods

### 2.1. Participants

The data acquired to assess reliability and concurrent validity were obtained from separate cohorts. The reliability cohort consisted of a convenience sample of 18 healthy individuals (11 female, 23.3±2.5 years, 168.2±11.2 cm, 78.2±17.8 kg). The validity cohort consisted of a convenience sample of 13 healthy individuals (eight females, 24.8±3.1 years, 170.0±7.7 cm, and 70.0±14.2 kg). Participants were included if they were between the ages of 18 and 35. Participants were excluded if they had sustained a lower extremity injury in the previous 6 months or if they had a history of vestibular disorders, balance disorders, or cardiac conditions or limitations. History of concussion was not considered exclusionary. The reliability and validity components of this study were approved by the university’s Institutional Review Board.

### 2.2. Instrumentation

**Figure 1:**
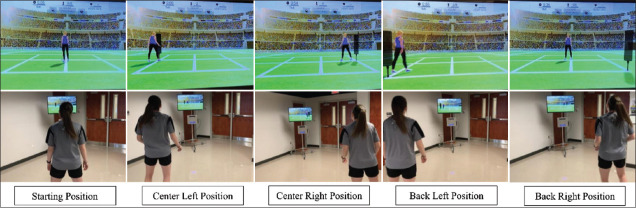
Depiction of TRAZER protocol. Top row is the screen as viewed by the participant. Bottom row is the participant reacting to the location of the virtual target.

TRAZER uses a depth-sensing Microsoft Kinect camera to create a three dimensional map of a 1.75 × 1.75 m capture area. Once within the field of view, a simulated person (avatar) appears on the monitor and mirrors the participant as they respond to visual targets randomly appearing on the perimeter of the capture area. Anatomical landmarks (e.g., joint centers) are determined with a randomized decision forest algorithm with a reported 1 ms latency [23-25]. Before the protocol, each participant stands in the center of the capture area facing the TRAZER television screen for a brief (~5 s) calibration, during which the Kinect camera recognizes and identifies the participant. Following calibration, a visual target randomly appears at one of eight possible locations on the perimeter of the capture area (forward, backward, left, right, forward left diagonal, forward right diagonal, backward left diagonal, or backward right diagonal) (Figure 1). Once the indicator appears, the participant moves as quickly as possible to the location. Once TRAZER detects the participant in the correct location, the indicator disappears, and the participant returns to the start position to prepare for the next repetition. The protocol consists of forty repetitions (five at each of the eight possible locations), each repetition entailing seven to eight feet of travel, and with a complete trial taking approximately 3 min to complete. TRAZER does not output raw data, but provides pre-defined performance metrics such as reaction time, average/maximum velocity, average/maximum acceleration, and deceleration, and total distance traveled (see Nyman, 2017a and https://trazer.com/science for comprehensive background information on TRAZER).

### 2.3. Procedures

Following written informed consent, height and weight were obtained from each participant. To measure reliability, operationally defined as test-retest consistency, participants were not digitized with any 3D markers or instrumentation. They attended data collection sessions on three consecutive days at similar times each day, during which each participant performed the TRAZER protocol. Based on pilot testing, 3 consecutive days were used to account for a possible learning effect on the 1^st^ day.

For validity, operationally defined as absolute agreement, each participant attended a single session in which a reflective marker placed on the S2 spinous process was digitized with Vicon Nexus (Vicon, Oxford Metrics, London, England) software. A static capture was recorded. Participants then performed the TRAZER protocol while being tracked concurrently with the TRAZER system sampling at 30 Hz and an 8-camera Vicon 3D Motion Capture system sampling at 60 Hz, consistent with previous validity studies that have used higher sampling rates for 3D motion capture [4,18,23].

### 2.4. Data handling

TRAZER obtains its metrics by tracking the “base of the spine,” which was operationalized as the digitized S2 spinous process marker. To obtain measurements from three-dimensional data, raw coordinates for the S2 marker were exported from Vicon Nexus. Data were processed in R (R Core Team (2013). R: A language and environment for statistical computing) using the packages “signal,” [26] “imputeTS,” [27] “zoo,” [28] and “purr” [29]. Raw marker coordinate data were interpolated and filtered with a 12Hz low-pass 4^th^ order Butterworth filter. The cutoff of 12 Hz was chosen after a residual analysis indicated 13 Hz to be the optimum cut point to maximize the signal to noise ratio. Total distance, maximum velocity, maximum acceleration, average velocity, average acceleration, average deceleration, and reaction time were the outcomes of interest. However, due to the proprietary nature of TRAZER calculations, only total distance, maximum velocity, and maximum acceleration were able to be confidently computed independently from TRAZER; thus, for validity, only these three variables were used. They were calculated as follows:

Total distance (m) = ∑❘*p*_i_ - *p*_i-1_❘, where *p* = position and *i* = capture frame Maximum velocity (m/s) =
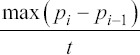
 , where *p* = position,

*i* = capture frame, and *t* = time Maximum acceleration (m/s^2^) = 
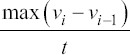
 , where *v* = velocity, *i* = capture frame, and *t* = time

### 2.5. Statistics

To assess reliability, a repeated-measures MANOVA was used to determine differences between days. Interclass correlation coefficients (ICC_(3, 3)_) and standard errors of the measurement (SEM) were computed for total distance, maximum velocity, maximum acceleration, maximum deceleration, average velocity, average acceleration, average deceleration, and reaction time. To assess validity, a repeated-measures MANOVA was conducted to compare means between the TRAZER and Vicon systems for total distance, maximum velocity, and maximum acceleration. Paired t-tests were conducted as post-hoc follow-ups. Pearson correlations, ICC_(2, 2)_, and SEM were also computed to determine levels of absolute agreement. ICCs were computed according to McGraw and Wong [30] and interpreted as follows: Less than 0.5, between 0.5 and 0.75, between 0.75 and 0.9, and greater than 0.9 were considered poor, moderate, good, and excellent, respectively [31]. Bland-Altman plots were constructed with the R package “BlandAltmanLeh” [32] to visually inspect validity and reliability data. Alpha was set at 0.05 for all statistical tests. Analyses were conducted in SPSS (IBM Corp, Armonk, NY).

## 3. Results

### 3.1. Reliability

A significant deviation from normality was evident for day one’s reaction time (Shapiro–Wilk *P*=0.002), but neither logarithmic nor square root transformation provided any substantial improvement in distribution normality. A repeated measures MANOVA including all eight dependent variables demonstrated a significant difference among trials (Wilks’ Lambda = 0.36; F_15,54_ = 2.23; *P*=0.015). With the exception of the three variables of average reaction time, average deceleration, and maximum acceleration, ICCs_(3, 3)_ across all three testing sessions were moderate to excellent, ranging from 0.65 to 0.95. Test-retest consistency among trials, Shapiro–Wilk test of normality result for each trial, and univariable repeated measure analysis of variance results for differences among trials are presented in Tables 1 and 2.

**Table 1:** Test-retest consistency among trials (ICC_3,3_), mean, standard deviation, and Shapiro-Wilk test of normality result for each trial (P_S-W_), unvariable repeated measure analysis of variance result for difference among trials (P_Trials_), and standard error of measurement (SEM).

Variable	3-Day ICC_3,3_ (SEM)	Days 1 & 2 ICC_3,3_ (SEM)	Days 2 & 3 ICC_3,3_ (SEM)	Trial 1		Trial 2		Trial 3		
		
				Mean±SD	P_S-W_	Mean±SD	P_S-W_	Mean±SD	P_S-W_	P_Trials_
Total Distance (m)	0.745 (3.36)	0.794 (3.02)	0.761 (3.25)	82.01±7.26	0.151	81.77±7.10	0.795	79.39±7.53	0.714	0.076
Average Velocity (m/s)	0.847 (0.03)	0.819 (0.03)	0.864 (0.03)	0.68±0.11	0.575	0.68±0.09	0.621	0.68±0.08	0.771	0.937
Average Acceleration (m/s^2^)	0.919 (0.13)	0.852 (0.03)	0.957 (0.10)	2.11±0.47	0.740	2.14±0.51	0.338	2.13±0.53	0.208	0.915
Average Deceleration (m/s^2^)	0.948 (0.07)	0.927 (0.08)	0.907 (0.09)	1.73±0.33	0.806	1.78±0.28	0.735	1.86±0.35	0.367	0.008
Maximum Velocity (m/s)	0.654 (0.06)	0.665 (0.06)	0.559 (0.07)	0.82±0.12	0.282	0.85±0.11	0.173	0.84±0.11	0.253	0.444
Maximum Acceleration (m/s^2^)	0.171 (0.51)	0.067 (0.54)	0.032 (0.55)	2.83±0.61	0.915	3.33±1.12	0.001	2.82±0.53	0.263	0.121*
Maximum Deceleration (m/s^2^)	0.212 (0.13)	0.255 (0.14)	-0.446 (0.14)	2.30±0.44	…464	2.61±0.77	<.001	2.36±0.42	0.742	0.222*
Reaction Time (ms)	0.536 (31)	0.447 (40)	0.493 (40)	320±101	0.002	326±41	0.291	316±26	0.946	0.773*

* Mauchly’s test of sphericity identified a significant difference among trials (P <.05); Greenhouse-Geisser df adjustment

**Table 2:** Mean, standard deviation, Shapiro-Wilk test of normality result (P_S-W_), Pearson correlation, and associated P value for measurements derived from different motion analysis systems.

Variable	TRAZER		Vicon		Pearson r	P	Spearman’s ρ	P
	
	Mean±SD	P_S-W_	Mean±SD	P_S-W_				
Total Distance (m)	89.46±17.85	0.108	108.59±24.47	0.015	0.961	<.001	0.956	<.001
Maximum Velocity (m/s)	1.00±0.12	0.312	2.46±0.52	0.004	0.808	<.001	0.663	0.014
Maximum Acceleration (m/s^2^)	4.32±0.47	0.903	1.34±0.42	0.778	-0.090	0.770	0.011	0.972

### 3.2. Validity

**Figure 2:**
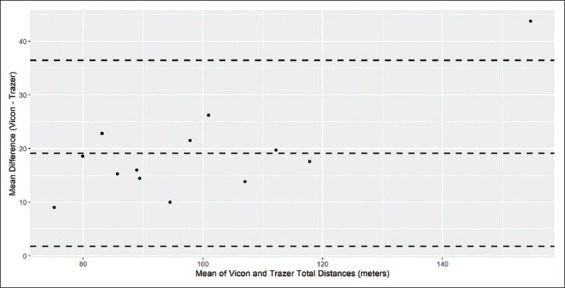
Bland-Altman plot depicting limits of agreement between TRAZER and three-dimensional motion capture for total distance. The central dotted line represents systematic error. The outer dotted lines represent ±2 standard deviations.

**Figure 3:**
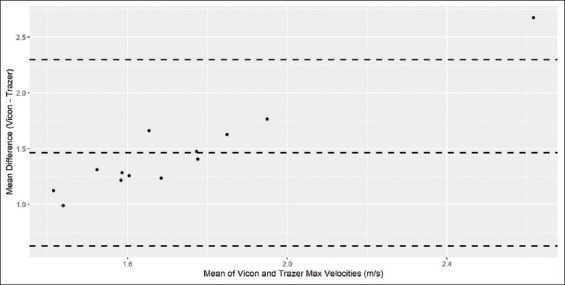
Bland-Altman plot depicting limits of agreement between TRAZER and three-dimensional motion capture for maximum velocity. The central dotted line represents systematic error. The outer dotted lines represent ±2 standard deviations.

**Figure 4:**
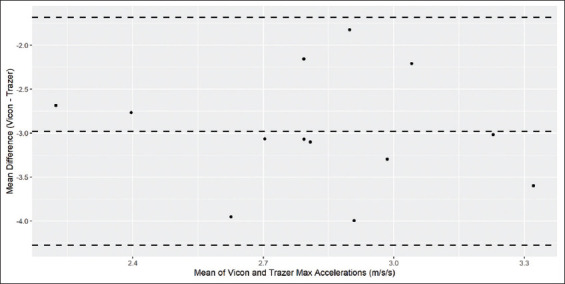
Bland-Altman plot depicting limits of agreement between TRAZER and three-dimensional motion capture for maximum acceleration. The central dotted line represents systematic error. The outer dotted lines represent ±2 standard deviations.

Vicon distributions for total distance and maximum velocity deviated significantly from normality, but neither logarithmic nor square root transformation provided any substantial improvements. Thus, the results of non-parametric correlations (Spearman’s rho) were also calculated and are presented in Table 2. A repeated measures MANOVA including all three dependent variables demonstrated a significant difference between systems (Wilks’ Lambda = 0.03; F_3,10_ = 94.00; *P*<0.001). Means, standard deviations, and Pearson r values for total distance, maximum velocity, and maximum acceleration obtained from TRAZER and Vicon are presented in Table 2. ICCs (SEM) for total distance, maximum velocity, and maximum acceleration were 0.79 (11.1m), 0.08 (0.5 m/s), and 0.01 (0.5 m/s^2^), respectively. Bland Altman plots are presented as visual representations of the absolute agreement between TRAZER and Vicon (Figures 2-4).

## 4. Discussion

We hypothesized that TRAZER would display moderate test-retest reliability and good to excellent concurrent validity. Our hypothesis was partially supported as evidenced by good to excellent reliability, but poor concurrent validity. To the best of our knowledge, such a comparison has not been previously performed. Although we observed a lack of concurrent validity between TRAZER and 3D motion capture, there was strong test-retest reliability of the TRAZER system particularly with regards to average acceleration, average velocity, and average deceleration.

Test-retest reliability of TRAZER was particularly strong with averaged measures such as average acceleration (ICC=0.919), average velocity (ICC=0.847), and average deceleration (ICC=0.948) in contrast to reliability of peak measures of acceleration, velocity, and deceleration (ICC range = 0.171–0.654). This is reasonable, as averaging a variable over the length of the TRAZER protocol is inherently more stable than extracting the peak from its path. These results align with Lopes’s meta-analysis [33], reporting high ICCs for 2D intra-rater reliability. Lopes’s meta-analysis reported an ICC of 0.99 for intra-rater reliability in studies that observed frontal plane projection angles with 2D video systems. Although this meta-analysis assessed joint angle reliability, while the current study inspected X-Y coordinate data, this lends further confidence to the reliability of 2D video systems such as TRAZER. As such, TRAZER may be appropriate for use in a setting in which serial measures (test-retest) are obtained to monitor progress, such as a physical therapy clinic or strength and conditioning facility. For instance, periodic measurements could be obtained following a lower extremity injury to quantify the extent to which a patient achieves superior functional performance. Furthermore, baseline TRAZER testing could be appropriate as a post-injury comparison to determine the point at which a patient reaches pre-injury level of function.

Observing high absolute agreement would have provided evidence to support the use of the more clinically-feasible TRAZER system as a clinical injury screening tool in comparison to the more burdensome gold standard 3D motion capture. The lack of strong concurrent validity between TRAZER and 3D motion capture is in stark contrast to a previous study (white paper) conducted by Nyman (Nyman, 2017). The findings of Nyman’s comprehensive evaluation testing TRAZER’s validity against 3D motion capture demonstrated high correlation for aggregate joint marker accelerations, decelerations, velocities, and distances (ICC range = 0.83–0.96). While both studies possessed similarities, the number of participants differed, Nyman had only two participants, whereas 18 participants were tested in the current study. For validation, we analyzed peak velocity and acceleration while Nyman reported average velocity and average acceleration. Average measures will be more stable than isolated peak measures and may explain the differences between the two sets of findings. Without access to TRAZER’s proprietary algorithms for calculation of these metrics, we were unable to assess TRAZER’s metric calculations and instead computed the metrics using data obtained from 3D motion capture. It should be noted that Nyman’s work was a white paper, for which raw coordinate data were available. As such, TRAZER time series trajectories of the S2 marker were overlaid on Vicon-obtained trajectories and then submitted to a validity analysis, whereas we assessed the validity of TRAZER-reported discrete metrics with metrics calculated with Vicon coordinate data. Although Nyman reported excellent validity ICCs for time series data, coupling this with the current data suggests that gross metrics (e.g., trajectories, averages) are more valid than discrete (e.g., peaks, maximums, and minimums) measures.

In addition, the systematic error as depicted in the Bland Altman plots for TRAZER and 3D motion capture validity showed the lack of between-system agreement. Over the course of the 40 repetition protocol, TRAZER systematically captured 19.13 m less in total distance traveled than 3D motion capture, equating to an 18% discrepancy. A possible explanation for lack of agreement could be the difference in capture rate. It should be noted that the Vicon system sampled data at 60 Hz. This, coupled with an array of eight cameras, naturally allows more accuracy in detecting peaks and maximums, compared with the TRAZER system operating with a single camera and a capture rate of 30 Hz. While TRAZER primarily picks up gross movements, Vicon is designed to record more finely tuned peaks that TRAZER may gloss over, thus producing unagreeable measures. This may explain the systematic differences between the two systems. The authors elected for a 60 Hz Vicon sampling rate because this is more representative of a gold-standard motion capture collection. This is consistent with other researchers who have used different sampling frequencies when comparing two-dimensional with three-dimensional motion capture [4,18,23]. In fact, in the white paper reported by Nyman, three-dimensional motion capture was sampled at 120 Hz, while TRAZER was sampled at 30 Hz. Thus, although different sampling rates likely partially explain the systematic differences observed between the TRAZER and Vicon in the current study, it does not fully account for the discrepancy.

### 4.1. Clinical and translational impact

A key advantage of the TRAZER system is the ability to closely replicate sport demands by presenting visual-cognitive virtual reality challenges that elicit quantifiable whole-body movement responses. Confidence in the system’s measurements of reactive responses for clinical documentation of pre- and post-injury performance capabilities and assessment of injury risk is supported by some of the validity and reliability coefficients derived from our testing. Exceptionally close agreement of the total distance measurement derived from TRAZER with that from the Vicon system, along with very good test-retest reliability; clearly support its use as an indicator of whole-body movement precision in the deactivation of virtual reality targets. These data indicate that TRAZER is appropriate for use as a baseline measure, in addition to post-injury quantification of return to pre-injury levels of functional capacity; thus, allowing for more individualized return-to-play protocols.

An important limitation of this study was the different sampling rates of each system; TRAZER captured at 30 Hz, while Vicon captured at 60 Hz. We acknowledge that this difference may partially account for discrepancies in accuracy. However, as gold standard three-dimensional motion capture typically samples at higher frequencies, this is a more externally valid comparison. Although participants were instructed to conduct similar activities prior to each day’s testing and were tested at similar times each day, we cannot discount the fact that outside factors may have influenced the participants’ energy levels and reactions each day. History of concussion was not considered exclusionary for participation. While this does not affect test-retest reliability or concurrent validity, the reader should caution against using these data as reflective of the larger population. Finally, validation efforts were significantly hampered by the inability to access proprietary algorithms utilized by TRAZER to calculate the mean data for validation. Relying on maximum outputs likely accounted for poor correlation across platforms with different capture rates and camera angles. As evidenced by a higher inter-rater reliability coefficient and r value for total distance, when measures are taken over time, instead of at maximal points in time, the systems display higher congruency.

TRAZER is designed to be clinically feasible, transportable, and user-friendly compared to the expensive, training-intensive, and sedentary 3D motion capture system. Although the poor validity of TRAZER against a gold standard 3D motion capture hinders that ability to directly compare values obtained from each system, lack of validation does not inhibit TRAZER’s ability to provide reliable test-retest measures. Findings for the test-retest reliability of the TRAZER system may prove beneficial in future studies to determine best uses of TRAZER system in a clinical setting. Additional studies focusing on examining the validity of mean values with a larger sample size could provide further evidence of research and clinical usefulness.
